# A new perspective on cardiovascular function and dysfunction during endurance exercise: identifying the primary cause of cardiovascular risk

**DOI:** 10.5114/biolsport.2024.134757

**Published:** 2024-04-09

**Authors:** Amine Souissi, Ismail Dergaa, Samia Ernez Hajri, Karim Chamari, Helmi Ben Saad

**Affiliations:** 1 Université de Sousse, Faculté de Médecine de Sousse, Hôpital Farhat HACHED, Laboratoire de Recherche (Insuffisance Cardiaque, LR12SP09), Sousse, Tunisie; 2 Primary Health Care Corporation (PHCC), Doha, P.O. Box 26555, Qatar; 3 High Institute of Sport and Physical Education of Ksar-Said, University of La Manouba, Tunis, Tunisia; 4 Naufar Wellness & Recovery Center, Doha, Qatar

**Keywords:** Cardiovascular responses, Catecholamine, Exercise, Inflammation, Heat stress, Oxidative stress, Platelet aggregation, Thermoregulation

## Abstract

Exercise mechanical efficiency typically falls within the range of approximately 20 to 25%. This means that a great part of the metabolic energy converted to generate movement is released as heat. Therefore, the rise in core temperature during endurance exercise in humans is proportional to generated work. Cutaneous vasodilation occurs when the core temperature threshold is reached. The rise in heart rate in response to thermal stress is a cardiovascular response that increases cardiac output and skin blood flow. The cardiovascular response during endurance exercise is a complex phenomenon potentially influenced by the involvement of nitric oxide in active thermoregulatory vasodilation. Excessive exercise can create high oxidative stress by disrupting the balance between free radicals’ production and scavenging, resulting in impaired cardiovascular function. The above considerations are related to the severity and duration of endurance exercise. The first focus of this narrative review is to provide an updated understanding of cardiovascular function during endurance exercise. We aim to explore the potential role of oxidative stress in causing cardiovascular dysfunction during endurance exercise from a fresh perspective. Additionally, we aim to identify the primary factors contributing to cardiovascular risk during strenuous prolonged exercise by highlighting recent progress in this area, which may shed light on previously unexplained physiological responses. To ascertain the effect of endurance exercise on cardiovascular function and dysfunction, a narrative review of the literature was undertaken using PubMed, ScienceDirect, Medline, Google Scholar, and Scopus. The review highlighted that high oxidative stress (due to high levels of catecholamines, shear stress, immune system activation, and renal dysfunction) leads to a rise in platelet aggregation during endurance exercise. Importantly, we clearly revealed for the first time that endothelial damage, vasoconstriction, and blood coagulation (inducing thrombosis) are potentially the primary factors of cardiovascular dysfunction and myocardial infarction during and/or following endurance exercise.

## INTRODUCTION

Prolonged exercise in hot conditions induces an important increase in body temperature that can result in impaired physical and mental performances in humans [1, 2]. The rise in core temperature depends upon exercise duration and/or intensity [3, 4]. Competition for blood flow develops between thermoregulatory and metabolic processes [5] and this may become problematic during conditions where cardiac output and arterial blood pressure are reduced due to dehydration and/or severe hyperthermia [6]. Blood flow to the active muscles is required to satisfy metabolic needs, while simultaneous blood flow to the skin ensures thermoregulatory control [5]. The major cardiovascular adjustment to heat stress is an increase in skin blood flow (SkBF) in response to the increase in cardiac output [7]. Severe heat stress conditions lead to a substantial increase in resting SkBF due to thermoregulatory vasodilation [8, 9]. However, during exercise, SkBF fails to reach high levels compared to the resting state, as active skeletal muscles require a significant increase in blood flow [7]. Furthermore, prolonged exercise under warm or neutral conditions is accompanied by a complex phenomenon known as “cardiovascular drift” [10]. Cardiovascular drift manifests as a gradual increase in heart rate over time, accompanied by a decrease in both stroke volume and mean arterial pressure [10]. Traditionally, it has been suggested that cardiovascular drift reflects cardiac fatigue [11], serving as a marker of cardiovascular dysfunction and/or limitation [7]. However, we have previously proposed a novel phenomenon that can potentially explain cardiovascular drift [10], extending beyond the realm of cardiovascular fatigue alone. We posited that cardiovascular drift, characterized by changes in the force-frequency relationship, may serve as a protective strategy against potential damage induced by strong myocardial contractions [10]. It is important to note that the focus of this current narrative review does not revolve around cardiovascular drift and dehydration. Instead, our aim was to provide an updated (until December 2023) understanding of the molecules involved in mediating cutaneous vasodilation during strenuous endurance exercise in the heat and/or normothermia, along with potential risk factors contributing to cardiovascular dysfunction and fatigue. The present narrative review of the literature was undertaken using PubMed, ScienceDirect, Medline, Google Scholar, and Scopus.

## MATERIALS AND METHODS

In this narrative review we searched databases including PubMed, ScienceDirect, Medline, Google Scholar, and Scopus for studies published up to 2023. The search focused on keywords such as “endurance exercise”, “cardiovascular function & dysfunction”, “thermoregulatory control”, “oxidative stress”, and “free radicals”. Inclusion criteria targeted studies relevant to the thermoregulatory control and cardiovascular responses to strenuous endurance exercise in the heat, particularly emphasizing the role of free radicals in cardiovascular function and dysfunction. Exclusion criteria encompassed non-English publications. Data extraction emphasized study design, participant details, exercise protocols, and cardiovascular measures. The quality of each study was assessed based on its design, methodology, and analytical rigor. Findings were synthesized to align with the review’s objectives, focusing on the cardiovascular responses to strenuous endurance exercise and the identified risk factors.

### Thermoregulatory control during exercise

During heat exposure and/or prolonged exercise, body temperature elevation induces a rise in the diameter of cutaneous blood vessels and therefore a reduction in vascular resistance [12]. Heart rate increases to restore peripheral blood pressure. Consequently, SkBF and skin temperature increase, which enhances heat dissipation via convection [8, 13]. Moreover, heat dissipation by evaporation of sweat needs heat transfer to the skin via cutaneous vasodilation [14, 15]. Indeed, the large increase in SkBF requires a significant elevation in cardiac output and a reduction of blood flow to renal and splanchnic circulations [16]. These adjustments could be sufficient to match the demand for increased SkBF at rest [8]. However, during prolonged exercise (of moderate or high intensity) in the heat or thermoneutral conditions [4, 17, 18], the heat loss remains inferior to heat generation. Therefore, in such conditions, the body cannot stop temperature elevation or maintain steady-state core temperature. Thus, the body temperature continues to rise even after an adequate thermoregulatory response, but at a lower rate compared to when prolonged exercise was started [17, 18]. The increase in core temperature depends on both exercise intensity and duration [3, 4]. It has been identified that power output capability and integrated electromyographic activity of the muscles decrease during prolonged exercise under heat stress [19, 20]. It has been reported that the neural recruitment of skeletal muscle motor units is reduced when core body temperature rises to “critical” levels [19, 20]. The critical temperature might be one of the major factors limiting muscular function [21]. Indeed, the efferent command to active muscles is attenuated when a high core temperature is reached (e.g. ∼39-40°C) [2]. The critical temperature may function as a signal for the central nervous system, which will then inhibit motor unit recruitment to protect the brain against severe hyperthermia [2, 20, 22, 23]. We confirm that the central nervous system may reduce neural drive and attenuate skeletal muscular function to prevent overheating. On the other hand, we suggest that the decrease in myocardial function cannot be considered as a strategy for preventing overheating as it may disturb the thermoregulatory control and increase the risk of heat stroke [17]. Interestingly, it is still unknown whether the decrease in cardiac performance *(i)* is simply due to an alteration in preload/afterload, and/or cardiac fatigue and/or cardiac damage, or *(ii)* it may be partially related to a protective strategy against the potential damage that could be induced by a permanent strong myocardial contraction [10].

### Cutaneous vasodilation during exercise

It is well known that cutaneous vasodilation is attenuated during exercise relative to the rest condition [5, 24]. Exercise initiation induces competition between the vasoconstrictor and vasodilator cutaneous systems [5]. Cutaneous vasoconstriction occurs in response to the initiation of exercise in heat-stressing conditions [5]. Exercise places several limits on the ability of the skin to dilate, due to an increased vasoconstrictor tone and decreased vasodilator capacity [5]. Furthermore, exercise influences the temperature threshold (which tends to rise) at which cutaneous vasodilation starts [5, 25]. Consequently, the central temperature increases brusquely in the first few minutes of exercise. Otherwise, despite further significant core temperature increases particularly in severe heat stress conditions, the rise in SkBF is attenuated when the central temperature approaches 38°C [26].

It has been reported in the literature that the exercise effect is intensity- dependent; low intensity does not alter thermoregulatory cutaneous vasodilation, whereas high intensity shifts the temperature threshold (which tends to rise) for cutaneous vasodilation [27–29]. However, the stimulus behind the exercise-induced shift in the temperature threshold for cutaneous vasodilation remains unclear [26], Interestingly, since it has been observed that cutaneous vasodilation during high-intensity exercise in the heat is attenuated relative to that during low-intensity exercise due to increased oxidative stress [30, 31], we speculate that oxidative stress is a potential factor explaining the shift in the temperature threshold.

There are several agents that have recently been found to act as vasodilators during exercise. Louie et al. [32] reported that ATP-sensitive potassium, calcium-activated potassium channels, and voltage- gated potassium channels contribute to the control of cutaneous vasodilation during exercise in heat stress conditions. Furthermore, Fujii et al. [33] highlighted that heat shock protein 90 contributed to cutaneous vasodilation during exercise via nitric oxide (NO) synthase (NOS)-dependent mechanisms. Importantly, Fujii et al. [33] also stated that NOS contributes to ∼40-50% of total cutaneous vasodilation during prolonged exercise in healthy humans. This finding reflects the major role of NOS in the thermoregulatory control (vasodilation response) during endurance exercise [33–36]. The vasodilation response to heat stress appears to be potentiated by NO production at rest and during exercise. Charkoudian et al. [37] identified that the cutaneous vasodilation response to a local warming (i.e. 30 minutes at 42°C) stimulus is biphasic and NO plays an important role in both the initiation and the maintenance of the second slower phase.

**FIG. 1:**
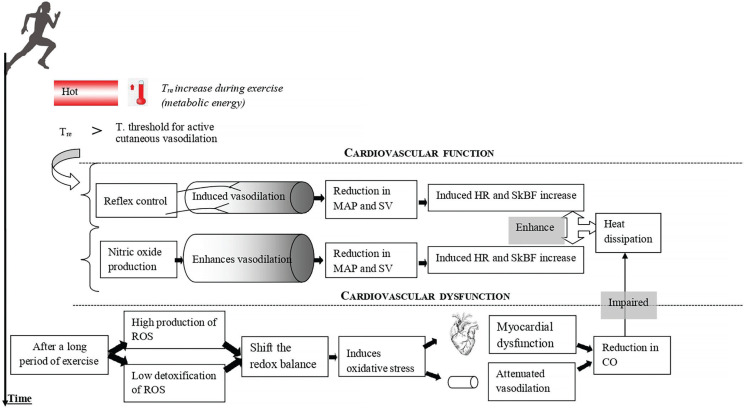
Simplified representation of the cardiovascular response to prolonged exercise. CO: cardiac output. HR: heart rate. MAP: mean arterial pressure. ROS: reactive oxygen species. SkBF: skin blood flow. SV: stroke volume. T: temperature. T_re_: rectal temperature.

Mechanisms mediating cutaneous vasodilation differ between whole-body and local skin heating [38]. However, it appears that there are some common points between them (e.g. NO-induced vasodilation) [33–36]. The skin vasodilation response to endurance exercise depends on reflex control (i.e. sensory nerve-mediated vasodilation) and free radicals (cutaneous endothelial NO-dependent vasodilation) (Figure 1). It has been demonstrated that skin vasodilation in heat stress depends on NO at rest and during exercise, with sensory nerves mediating an initial transient vasodilatory “peak” followed by a prolonged vasodilatory “plateau” mediated primarily by NO production [33, 39, 40]. We highlight that it is still unknown whether the source of NO during “local heating” is the same as the one released during exercise where the heat generation comes from the muscular work. Future research should pay more attention to such issues.

### Cardiovascular fatigue related to oxidative stress during endurance exercise

Multiple factors contribute to cardiovascular fatigue during prolonged exercise and make it a complex phenomenon [41–43]. Meta-analyses revealed a reduction in left ventricular function following 24-h exercise [42, 43]. During prolonged exercise, the large displacement of blood flow toward the skin appears to play a key role in the development of cardiovascular fatigue [17].

Exercise results in an increase in heart rate, which increases the mechanical forces of blood flow on the vascular wall (i.e. shear stress and blood pressure) [44]. Shear stress has been shown to increase endothelial superoxide generation in conductance arteries in vivo [45]. Similarly, high levels of shear stress during prolonged exercise have been found to stimulate vascular superoxide and hydrogen peroxide production [46]. Exercise-induced oxidative stress has recently been well confirmed [47–51], and it is well established that the increased production of reactive oxygen species (ROS) during exercise has both positive and negative physiological effects [47, 51–53]. The latter may shift the redox balance to a pro-oxidant state and impair thermoregulatory vasodilation [47, 49, 51]. According to this fact, studies indicated that NOS-dependent cutaneous vasodilation during high-intensity exercise in the heat is attenuated relative to that during moderate-intensity exercise due to increased oxidative stress [30, 31].

Nicotinamide adenine dinucleotide phosphate oxidase enzyme (NADPH) is the major source of ROS in the heart [54, 55], and also well known for its role in myocardial dysfunction [55]. Interestingly, a significant link between NADPH oxidase-dependent oxidative stress and myocardial dysfunction has been identified after prolonged strenuous exercise in rats [56–58]. This might potentially represent a new trigger in the understanding of exercise-induced myocardial dysfunction. High oxidative stress was proposed to depress cardiac function through protein kinase G and cyclic monophosphate-mediated desensitization of cardiac myofilaments [59].

Furthermore, a long period of exercise increases catecholamine and pro-inflammatory markers such as tumour necrosis factor and interleukin- 6 (IL-6) [60–62]. Excess catecholamines contribute to an increase in ROS formation [61], and attenuation of β-adrenergic inotropic responsiveness [63–66]. A previous study highlighted that cardiac stress induced by exercise activates both oxidative stress, inflammation and β-adrenergic pathways [67]. It is well known that oxidative stress and inflammation are potentially involved in the pathogenesis of heart failure and impaired left ventricular function [56–58, 68, 69]. Interestingly, the administration of IL-1 and tumour necrosis factor is associated with a decline in contractile function in rats’ hearts, this being partially improved by treatment with an NOS inhibitor [70]. Moreover, antioxidant supplementation may attenuate the decline in myocardial function during exercise [67, 71, 72].

### Does inflammation cause cardiovascular risk during endurance exercise?

**FIG. 2:**
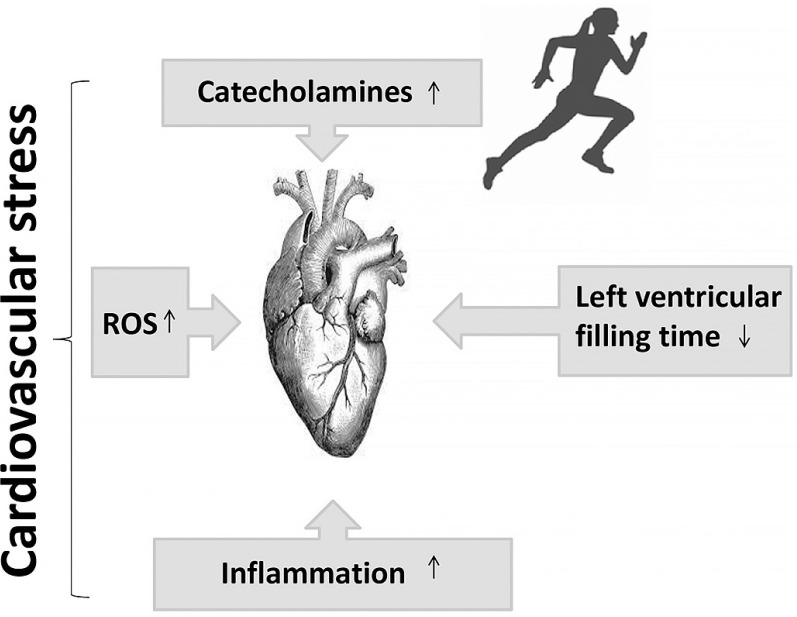
Putative mechanisms leading to cardiovascular stress during exercise. ROS: reactive oxygen species.

For a considerable period, the notion of cardiovascular risk has been associated with increased cardiovascular stress leading to alterations (decline) in cardiovascular function [12, 17] (Figure 2). Recent studies have attributed the decline in cardiac function during exercise to adrenergic desensitization (βj and β_2_ receptors), [10, 73, 74], which attenuates cardiac function and provides cardioprotection against acute stressors induced by exercise, such as mechanical stress, cellular damage, and mitochondrial damage [10, 42, 75]. Interestingly, alongside the controlled mechanisms of β-adrenergic receptor desensitization and β_3_ activation, high levels of oxidative stress linked to inflammation [10, 62, 76, 77] may also contribute to the attenuation of cardiac function.

In fact, studies have demonstrated that inhibition of endogenous NO leads to an enhancement of the effects of catecholamines [78–80], indicating that NO may play a cardio-protective role by attenuating the effect of catecholamines, potentiating the positive inotropic effect in normal hearts. Accordingly, it has been confirmed that the increase in NO concentrations attenuates the positive inotropic effect of catecholamines [81]. Furthermore, certain molecules associated with inflammation, such as IL-10, brain-derived neurotrophic factor, and follistatin-like protein, which increase during or after exercise, may also play a cardio-protective role by facilitating tissue repair and regeneration [82]. These findings raise a crucial question regarding the initial causes of cardiovascular risk. The primary focus of the present narrative review is to elucidate our current understanding of cardiovascular dysfunction during prolonged exercise with respect to inflammation and oxidative stress. Additionally, we aim to identify the primary cause of cardiovascular risk.

### Cardio-renal association and immune response activation Renal vasoconstriction and NADPH oxidase activation

The large increase in SkBF during endurance exercise requires a significant reduction of renal blood flow [16]. The decrease in renal blood flow [16] constitutes a potential factor for the decline in renal function. Sustained levels of sympathetic activity and dehydration may contribute to the increase in plasma renin activity and circulating angiotensin II [83, 84]. Then, the activation of angiotensin II type 1 receptors *(i)* elevates ROS production through NADPH oxidase activation [85–87], and *(ii)* stimulates aldosterone secretion [88, 89]. In turn, aldosterone can directly stimulate ROS production through the activation of NADPH oxidase [90]. Importantly, the stimulation of aldosterone release is linked to an increase in galectin-3 concentration in the heart and kidney [77, 91]. Galectin-3 and other markers of cardiac damage (e.g. troponin I, suppression of tumorigenicity 2 (ST2)) increase following strenuous physical effort [92, 93]. Interestingly, as at December 2023, there remains no explanation of the reason for this increase, which has been suggested to be physiological [94, 95]. This narrative review seeks to clarify our comprehension of the factors contributing to the elevation of galectin-3 levels in reaction to strenuous endurance exercise.

### Galectin-3 and inflammatory biomarkers

When cardiovascular tissues are physically damaged, the immune system is activated in order to remove the damaged cells and maintain homeostasis in the body [96]. Galectin-3 seems to play an important role in immune response activation [97] by stimulating hyperoxide secretion from neutrophils through NADPH activation [98, 99]. Neutrophils are generated and sent to the site of injury within minutes and are the hallmark of acute inflammation [100]. Suzuki [101] highlighted the protective role of neutrophils in the exercise-induced muscle damage associated with high production of ROS. Hence, neutrophil levels have been shown to double during prolonged strenuous exercise and remain elevated for at least 24 h [102].

Galectin-3 is actually considered a reasonable cardiovascular inflammatory biomarker [97]. Although galectin-3 is identified as a risk predictor of adverse events and cardiac arrest [103, 104], its levels correlate also with tissue repair [105, 106]. Galectin-3 should not be considered a pathogenic molecule inducing cardiovascular damage, since it contributes to activation of the immune system, playing a cardioprotective role during exercise. Interestingly, it was observed that the blood levels of galectin-3 in endurance athletes were more elevated than in sedentary healthy humans at the beginning and the end of exercise [107], confirming that galectin-3 can play a cardioprotective role in athletes. A 2020 review suggested that galectin-3 could be used as a novel treatment for atherosclerosis [97]. In fact, galectin-3 can play an anti-inflammatory role and exert a beneficial effect on atherosclerosis by activating M2 macrophage differentiation, via the CD98/ phosphoinositide 3-kinase pathway [108]. On the other hand, we highlight that a clinical 2022 study found that galectin-3 accumulation potentiates platelet aggregation via dectin-1 activation [109], which can promote thrombosis during/or following exercise.

### Aldosterone, fibroblast growthfactor 23 and parathyroid hormone

It is well established that fibroblast growth factor 23 (FGF-23), a newly discovered hormone, and parathyroid hormone (PTH) are markers of chronic kidney injury and cardiovascular impairment (i.e. ventricular hypertrophy, vascular calcification, and arterial stiffness) [110]. The literature revealed that high-intensity endurance exercise training is associated with arterial stiffness [111], ventricular hypertrophy [112], and coronary artery calcification [113, 114]. Importantly, the increase in PTH level depends on exercise severity (intensity and duration), suggesting a complex cause-and-effect relationship between PTH and coronary artery calcification [113]. Currently (December 2023), the mechanism explaining PTH elevation during exercise is not yet well understood. In fact, RAS is activated during prolonged exercise [115], indicating a decline in renal function and then an impairment in calcium and phosphate regulation. Therefore, PTH and FGF-23 are both secreted to restore calcium and decrease phosphate levels [116].

**FIG. 3:**
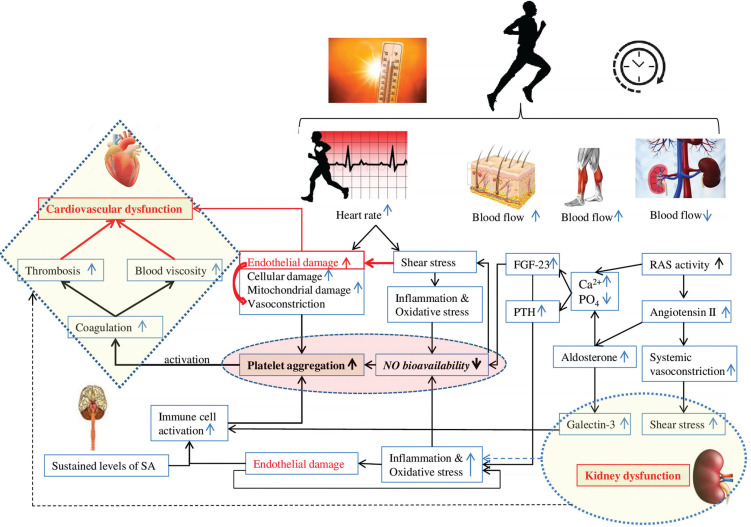
Potential factors contributing to coagulation and cardiovascular dysfunction during prolonged exercise. FGF-23: fibroblast growth factor 23. PTH: parathyroid hormone. RAS: renin-angiotensin-aldosterone system. SA: sympathetic activity.

We hypothesize that aldosterone, PTH and FGF-23 play a key role in the development of cardiovascular fatigue during exercise: Aldosterone increases oxidative stress, reduces NO levels, and promotes platelet adhesion possibly by potentiating collagen production [117, 118], triggering a cascade of coagulation. We believe that aldosterone can play a crucial role in the development of thrombosis, resulting in cardiovascular dysfunction during and following endurance exercise. FGF23 directly alters endothelial vasodilation function by reducing NO bioavailability [119] (Figure 3). Importantly, clinical studies identified a strong correlation between PTH elevation and hypercoagulability [120]. One could speculate that PTH can contribute to blood coagulation and cardiovascular dysfunction during prolonged exercise possibly by affecting calcium levels and potentiating oxidative stress [121].

### Toll-like receptor 4 (tlr4) and coagulation

The analysis of TLR4 is a promising advanced orientation in the search for understanding cardiovascular dysfunction [122]. Indeed, a single bout of exercise induces TLR4 activation, promoting endoplasmic reticulum stress [122, 123], an inflammatory response [122–124], and increased markers of cardiac injury [122, 123] and apoptosis [122] (i.e. heart-damaging events), which are necessary mechanisms for remodelling and adaptation [122]. Interestingly, it has been recently suggested that nuclear factor- kappa B (NF-kβ) activation by TLR4 is a necessary mechanism for cardiovascular adaptations induced by physical effort [122]. According to Gordon et al. [125], NF-kP can promote desirable adaptive responses. Cardioprotection is triggered during or immediately after exercise when cells are under oxidative stress [126]

**FIG. 4:**
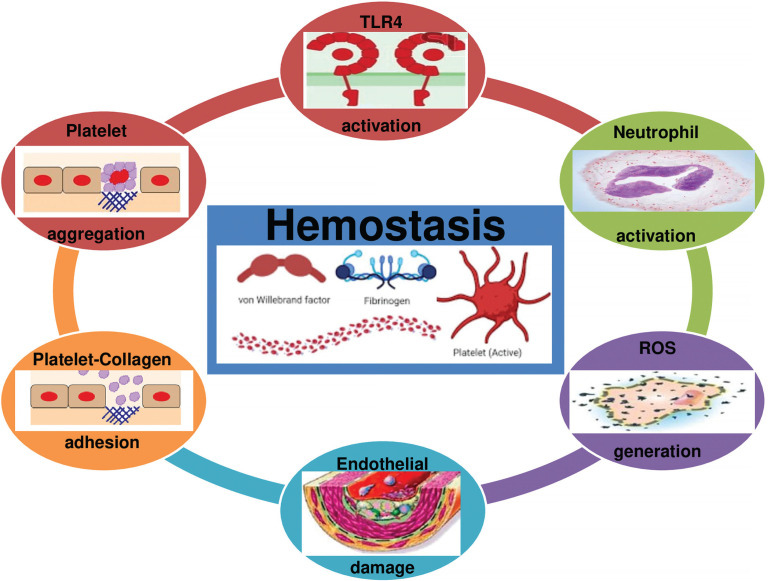
Toll-like receptor 4 (TLR4) promotes acute coagulation via neutrophil activation, ROS generation and platelet aggregation. ROS: Reactive oxygen species.

Although TLR4 is a physiological response favouring apoptosis and tissue regeneration to assure beneficial adaptive responses to exercise, evidence suggests that TLR4 promotes acute coagulation [127] via NF-kβ activation, neutrophil activation, ROS generation, and platelet aggregation [127, 128] (Figure 4). Therefore, we highlight that TLR4 activation by acute strenuous exercise can result in undesirable cardiac events (myocardial infarction or sudden death), particularly in people with cardiovascular diseases [41].

### What is the primary cause of cardiovascular risk?

To date (December 2023), inflammation has traditionally been regarded as the primary cause of cardiovascular dysfunction during endurance exercise. However, there is abundant evidence indicating that the inflammatory response is not the primary cause of dysfunction [129]. Importantly, some anti-inflammatory cytokines (e.g. IL-10) inhibit activation of coagulation and regulate thrombosis [130, 131]. Furthermore, it is well established that the IL-33/ST2 axis exhibits a cardioprotective role, reducing fibrosis and cardiomyocyte hypertrophy, and improving myocardial function in both chronic and acute heart failure [132]. Moreover, creatine kinase is traditionally considered an indicator of tissue damage and subsequent fatigue. Creatine kinase’s function is intriguing. Its role in cardioprotection, particularly through the inhibition of ADP-triggered platelet activation [133], underscores the imperative for researchers to recalibrate and deepen their perspectives on the multifaceted biological responses to exercise.

White blood cells and platelet counts are reported to be elevated after prolonged strenuous exercise [134–136]. It was reported that inhibition of platelet aggregation by NO was decreased following acute prolonged strenuous exercise. The attenuated response of platelets to NO during exercise results in thrombotic complications [137]. Moreover, NADPH plays a crucial role in ROS generation and platelet activation [138]. Indeed, such activation despite its protective role through haemostasis-inducing coagulation can result in thrombosis, myocardial infarction, and cardiac arrest [139].

**FIG. 5:**
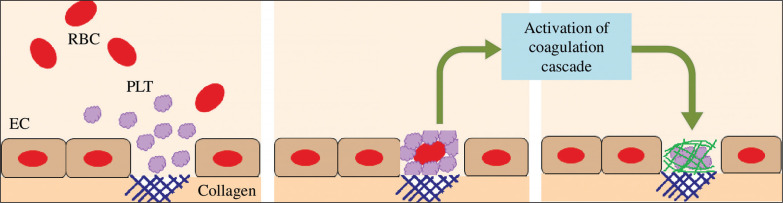
Platelets are activated when binding to the collagen substratum, which promotes a cascade of coagulation. EC: endothelial cell. PLT: platelet. RBC: red blood cell.

Moreover, it is well established that strenuous exercise induces endothelial damage/vascular injury [10]. The damage of endothelial cells results in vasoconstriction and exposes the collagen substratum to the blood circulating at high shear rates [128]. Therefore, platelets circulating at high velocity will adhere to collagen. The adherent platelets then aggregate. Platelets are then activated (by secreting ADP, serotonin and TxA2) which promotes a cascade of coagulation [128] (Figure 5). Therefore, endothelial damage can further trigger haemostasis, resulting in a high level of blood viscosity, potentiating thrombosis and cardiovascular dysfunction during and/or following exercise.

In summary, our review suggests that the primary causes of cardiovascular risk during acute endurance exercise are two-fold. Firstly, cell damage (involving endothelial and mitochondrial cells) emerges as a significant contributor. The stress imposed during exercise can lead to damage in these cells and result in vasoconstriction, potentially impacting cardiovascular function and overall risk. Secondly, blood coagulation (due to cell damage, inflammation, renal dysfunction, and high oxidative stress) leading to thrombosis and an increase in blood viscosity, plays a crucial role in cardiovascular risk. These factors can affect blood flow dynamics and potentially lead to adverse cardiovascular events. Additionally, it is important to consider coronary artery calcification as a risk factor that may be exacerbated after high-load training. This calcification process can further contribute to the overall cardiovascular risk profile.

In conclusion, the combination of several factors –*(i)* shear stressinduced endothelial damage promoting coagulation cascade and then thrombosis, *(ii)* oxidative stress, inflammation, renal dysfunction, and immune activation promoting blood coagulation and then thrombosis, and *(iii)* vasoconstriction (due to endothelial damage, oxidative stress, and haemostasis) promoting thrombosis – contributes to the increased cardiovascular fatigue and risk (myocardial infarction) during and/or following strenuous endurance exercise. Finally, we highlight that dehydration can potentiate cardiovascular fatigue and the risk of cardiovascular infarction.

### Perspective and limitations

Our hypothesis revolves around the notion that excessive oxidative stress, potentially linked to shear stress, cellular damage, renal dysfunction, and activation of the immune response, contributes to cardiovascular dysfunction through the promotion of platelet aggregation. This hypothesis is firmly grounded, supported by compelling evidence indicating that vigorous exercise leads to a significant rise in platelet aggregation and activation [140, 141]. Additionally, there exist various other molecules associated with impaired energetic metabolism and cardiovascular dysfunction (such as free fatty acids, cholesterol, low-density lipoprotein, catecholamines, and serotonin) [142] that may further exacerbate coagulation during and/or following exercise [143, 144]. Investigating the complex association of these variables with coagulation warrants comprehensive exploration in future studies. Of particular interest, the use of antithrombotic therapy holds promising potential in counteracting coagulation-induced cardiovascular fatigue. However, caution must be exercised due to the inherent risk of bleeding complications. Markers of cardiac/renal damage (e.g. galectin-3) increase during and/or following exercise; however, there has been no explanation of the reason for this response, which has been suggested to be physiological (i.e. non-pathological), though it remains unexplained to date. Notably, this increase in markers is potentially associated with activation of TLR4. TLR4 activation promotes an inflammatory response and increases markers of cardiac injury, which are crucial for remodelling. Immune activation and inflammatory responses to acute exercise are both adequate physiological responses, ensuring a cardioprotective role by optimizing cardiovascular adaptation to physical effort (i.e. training). However, excessive immune system activation and inflammation during exercise are associated with a heightened risk of cardiac events. Although evidence suggests that galectin-3 is secreted to promote physiological cardiac remodelling and immune system activation, it is not yet clear whether galectin-3 contributes to cardiovascular dysfunction by promoting platelet activation and thrombosis (i.e. by activating dectin-1/spleen tyrosine kinase signalling). This narrative review serves as a call to researchers to explore and investigate this potential pathway for a better understanding of cardiovascular risk prevention through exercise.

The present narrative review, while providing valuable insights, does have additional perspectives. We acknowledge that not all biomarkers of inflammation (e.g. IL-6 and IL-17), iron deficiency, and numerous other factors potentially exacerbating coagulation during exercise have been discussed. Furthermore, our focus centred on oxidative stress during exercise, while neglecting the influence of other factors, including alterations in pH, calcium levels, and energy metabolism, which can also contribute to cardiovascular dysfunction [145]. We encourage further exploration in this field to enhance our understanding of cardiovascular dysfunction during prolonged exercise by incorporating additional information and complementing our current knowledge base.

## CONCLUSIONS

The thermoregulatory vasodilation during prolonged exercise primarily relies on reflex control and nitric oxide production. However, extensive evidence from the literature indicates that cutaneous vasodilation during strenuous exercise is impaired by excessive ROS production and increased oxidative stress. Furthermore, reducing the inhibitory effects of nitric oxide on platelet aggregation during prolonged strenuous exercise may result in potentially dangerous thrombotic complications.

Prolonged strenuous exercise has been shown to increase NADPH oxidase activity, shift the redox balance to a pro-oxidant state, and impair renal and cardiovascular function. NADPH plays a critical role in the formation of ROS, as well as immune and platelet activation. Although such activation serves an immune-protective function, it can lead to coagulation and myocardial infarction.

## Declaration

In order to correct and improve the academic writing of our paper, we have used the language model ChatGPT 3.5 [146, 147].
